# Draft Genome Sequences of Five *Legionella pneumophila* Strains Isolated from Environmental Water Samples

**DOI:** 10.1128/genomeA.00474-15

**Published:** 2015-05-14

**Authors:** Kenta Watanabe, Haruo Suzuki, Ryo Nakao, Takashi Shimizu, Masahisa Watarai

**Affiliations:** a The United Graduate School of Veterinary Science, Yamaguchi University, Yamaguchi, Japan; b Joint Faculty of Veterinary Medicine, Laboratory of Veterinary Public Health, Yamaguchi University, Yamaguchi, Japan; c Department of Environmental Science and Engineering, Graduate School of Science and Engineering, Yamaguchi University, Yamaguchi, Japan; d Unit of Risk Analysis and Management, Hokkaido University Research Center for Zoonosis Control, Sapporo, Hokkaido, Japan

## Abstract

*Legionella pneumophila* is the causative agent of legionellosis. Here, we report the draft genome sequences of five *L. pneumophila* strains, Bnt314, Ofk308, Twr292, Ymg289, and Ymt294, isolated from environmental water samples. Comparative analyses of these genomes may reveal the survival mechanisms and virulence of *L. pneumophila* in the natural environment.

## GENOME ANNOUNCEMENT

*Legionella pneumophila* is the most frequent cause of legionellosis in humans. *L. pneumophila* can withstand temperatures of 0 to 68°C and a pH range of 5.0 to 8.5 and can survive in most environments for long periods (1). Furthermore, *L. pneumophila* is a facultative intracellular bacterium and can survive within free-living protozoa, such as amoebae and ciliates, in environmental waters (2–4). These environmental habitats of *L. pneumophila* have been suggested as important evolutionary sites to develop their virulence traits in humans, although the detailed mechanisms remain largely unclear.

Previously, we isolated five *L. pneumophila* strains from environmental water samples (5). *L. pneumophila* strains Ofk308, Twr292, and Ymt294 were isolated from an Ashiyu foot spa strain Ymg289 was isolated from a water fountain, and strain Bnt314 was isolated from a pond. These strains were classified as *L. pneumophila* serotype I or IV. Here, we report the draft genome sequences of the five *L. pneumophila* strains (Table 1).

**TABLE 1:** Information for draft genome sequences of five *L. pneumophila* strains isolated from environmental water samples

Strain	Source	Serotype	Accession no.	No. of contigs (>200 bp)	G+C content (%)	Genome size (bp)	No. of protein-coding genes
Bnt314	Pond	IV	BBUG00000000	80	38.2	3,471,799	3,105
Ofk308	Ashiyu foot spa	IV	BBUH00000000	86	38.2	3,473,188	3,104
Twr292	Ashiyu foot spa	I	BBUI00000000	66	38.2	3,394,434	3,007
Ymg289	Water fountain	I	BBUJ00000000	54	38.3	3,689,833	3,291
Ymt294	Ashiyu foot spa	I	BBUK00000000	141	38.4	3,401,814	3,076

Chromosomal DNA was extracted from an overnight culture of the five *L. pneumophila* strains using the DNeasy blood and tissue kit (Qiagen). Whole-genome sequencing of the five environmental strains was performed using paired-end sequencing on the Illumina MiSeq kit version 3. The sequencer produced 300-bp paired-end reads that were obtained from 550-bp inserts. The quality of the reads was checked using FastQC (http://www.bioinformatics.babraham.ac.uk/projects/fastqc/), and the raw sequences were trimmed to 250 bp using FASTX-Toolkit (http://hannonlab.cshl.edu/fastx_toolkit/). *De novo* genome assembly was performed using SPAdes version 3.5.0 (6). After the removal of low-coverage contigs, the resulting contigs were ordered against the complete genome of *L. pneumophila* subsp. *pneumophila* strain Philadelphia 1 chromosome (7) using the Mauve aligner (8). Genome annotation was performed using Prokka version 1.11 (9). The genome sizes and G+C contents were estimated for all contigs of each strain using G-language Genome Analysis Environment version 1.9.0 (http://www.g-language.org) (10). Among the five strains, the genome size and G+C content varied from 3.39 to 3.69 Mb and 38.2 to 38.4%, respectively (Table 1) and were close to those of the reference genome of strain Philadelphia 1. The genome statistics for the five environmental strains are summarized in Table 1.

### Nucleotide sequence accession numbers. 

The sequences have been deposited as whole-genome shotgun projects at DDBJ/EMBL/GenBank under the accession numbers listed in Table 1. The versions described in this paper are the first versions.
